# Ultrasensitive, Specific, and Rapid Detection of *Mycoplasma pneumoniae* Using the ERA/CRISPR–Cas12a Dual System

**DOI:** 10.3389/fmicb.2022.811768

**Published:** 2022-05-13

**Authors:** Zhongliang Deng, Haiyang Hu, Dan Tang, Jiaxin Liang, Xiaoling Su, Tingqing Jiang, Xipan Hu, Wanqin Ying, Deshuai Zhen, Xilin Xiao, Jun He

**Affiliations:** ^1^The Affiliated Nanhua Hospital, Department of Clinical Laboratory, Hengyang Medical School, University of South China, Hengyang, China; ^2^Department of Public Health Laboratory Sciences, College of Public Health, Hengyang Medical School, University of South China, Hengyang, China; ^3^Hunan Key Laboratory of Typical Environmental Pollution and Health Hazards, College of Public Health, Hengyang Medical School, University of South China, Hengyang, China

**Keywords:** *Mycoplasma pneumoniae*, CRISPR-Cas12a, rapid detection, visualized detection, ERA

## Abstract

Mycoplasma pneumoniae can cause severe respiratory tract infections and extrapulmonary diseases, which pose a significant threat to the health of children. Diagnostic methods for *M. pneumoniae* include isolation and culture, antibody detection, fluorescence quantitative PCR, and so on, but there are various shortcomings in time, cost, convenience, and sensitivity. In this study, we developed a rapid, sensitive, specific, and economical method for the detection of *M. pneumoniae*, termed the ERA/CRISPR–Cas12a dual system. The system used the high specificity and collateral cleavage activity of the LbCas12a protein, combined with enzymatic recombination amplification (ERA) technology with strong amplification ability, allowing the results to be observed by a portable fluorometer or visualized by the naked eye with a dipstick, which could be obtained in approximately 30 min. The ERA/CRISPR–Cas12a fluorescence and dipstick system were able to detect *M. pneumoniae* at titers as low as 1 and 100 copies/μL, respectively. The specificity of the two interpretation methods was 100%, and no cross-reaction with other pathogens was observed. In the evaluation of 92 clinical samples, the positive predictive agreements of the ERA/CRISPR–Cas12a fluorescence and dipstick systems with qPCR detection were 100% and 92.86%, respectively. The negative predictive agreements of both methods were 100%. In conclusion, this study established a portable, rapid, low-cost, ultrasensitive, and specific method for the early and rapid diagnosis of *M. pneumoniae* to meet the needs of on-site rapid detection in primary health institutions.

## Introduction

*Mycoplasma pneumoniae* (*M. pneumoniae*) is the smallest prokaryotic microorganism that lacks a cell wall and is highly polymorphic. It can grow in an artificial medium (Himmelreich et al., 1996) and is a common pathogen that causes atypical pneumonia and community-acquired pneumonia (CAP). The clinical symptoms are mainly coughing, fever, sore throat, and muscle pain (Jiang et al., 2021), which are similar to other respiratory pathogens, such as the influenza virus and *SARS-CoV-2*, and most likely lead to misdiagnosis, which can delay treatment and lead to complications, such as respiratory failure and respiratory distress syndrome (Yang et al., 2019). In addition to causing respiratory diseases, *M. pneumoniae* can induce a variety of *M. pneumonia*-related extrapulmonary diseases (MpEPDs), such as liver involvement, Kawasaki disease, Henoch–Schonlein purpura, Stevens–Johnson syndrome, and crescentic glomerulonephritis (Poddighe, 2018, 2020), which pose a serious health threat to children and young people, so it is necessary to improve *M. pneumoniae* detection. The identification of *M. pneumoniae* includes isolation and culture, immunological diagnosis, and molecular diagnosis. The real-time PCR is a primary technology for early rapid detection of *M. pneumoniae* nucleic acid, which yields absolute and relative quantification with higher sensitivity and better specificity than ordinary PCR (Schmitt et al., 2013; Winchell and Mitchell, 2013), but has high requirements in terms of instrument, environment, and laboratory personnel (Shi et al., 2019; Wang et al., 2019b). To realize the early and rapid diagnosis of *M. pneumoniae*, it is urgent to establish a rapid, simple, sensitive, and specific field detection method, which is critical for the treatment of *M. pneumoniae* and the epidemic control of CAP.

Clustered regularly interspaced short palindromic repeats (CRISPRs) with CRISPR-associated (Cas) proteins are immune defense systems used by prokaryotes to defend against invasion by foreign genetic material (Liu et al., 2017; Wang et al., 2019a). The CRISPR–Cas9 system is widely used in gene engineering, whereas Cas12 and Cas13 proteins are more suitable for gene detection applications because of their simplicity and ease of use (Liu et al., 2017). On this basis, a variety of gene detection methods have been established, the most representative being Specific High-Sensitivity Enzymatic Reporter UnLOCKing (SHERLOCK) (Gootenberg et al., 2017), DNA endonuclease-targeted CRISPR trans reporter (DETECTR) (Chen et al., 2018), one-HOur Low-cost Multipurpose highly Efficient System (HOLMES) (Li et al., 2018), and others. These systems are based on Cas12/13-associated CRISPR arrays that are processed into mature guide RNAs (gRNAs) that can bind to Cas proteins into complexes, which then efficiently and specifically cleave the target sequence through a short protospacer adjacent motif (PAM). When activated, the Cas protein generates “collateral cleavage” activity, which can non-specifically cleave other oligonucleotide sequences (Zetsche et al., 2015). However, CRISPR systems have low sensitivity for detecting pathogens directly and cannot meet the demands of clinical testing. Therefore, the CRISPR–Cas system is typically combined with nucleic acid technology to detect pathogens. In recent years, a variety of isothermal amplification techniques have been developed, such as loop-mediated isothermal amplification (LAMP) (Notomi et al., 2000) and recombinase polymerase amplification (RPA) (Piepenburg et al., 2006). Compared with traditional qPCR, such methods can react at a constant temperature and have the advantages of simple instrumentation, short reaction time, and simple operation. Therefore, the combination of the CRISPR system with isothermal amplification technology has both improved the sensitivity and reduced the use of large instruments such as qPCR instruments, which provide a new prospect for pathogen detection.

**FIGURE 1:**
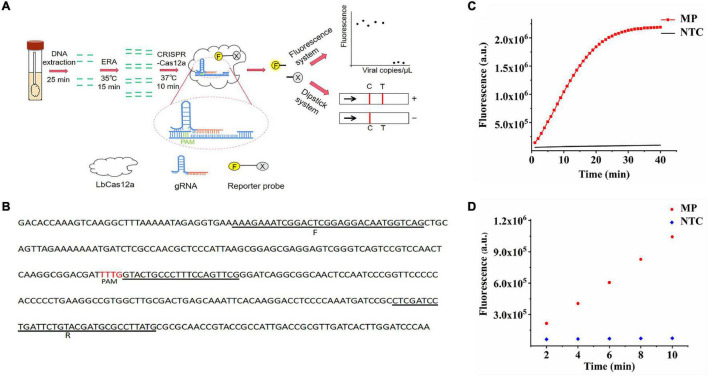
Feasibility of the ERA/CRISPR–Cas12a system. **(A)** ERA/CRISPR Cas12a dual-system framework. The DNA of *M. pneumoniae* samples was extracted within 25 minutes, and the target sequence was amplified by ERA (15 minutes). The LbCas12a-gRNA complex recognized the amplification product and triggered the “collateral cleavage” activity, which cleaved ssDNA reporters (15–20 min). Qualitative analysis can be carried out by observing the fluorescence signal or chromatographic dipstick. T: test line; C: control line. **(B)** The selected sequence was the target fragment of the *M. pneumoniae* adhesion *P1* sequence in this study. F was the forward primer of the ERA/CRISPR–Cas12a dual system, R was the reverse primer, PAM was the protospacer adjacent motif of the CRISPR array, and the immediately followed scribed fragment was the reverse complementary fragment of gRNA, which was the target sequence. **(C)** Real-time detection of Cas12a trans-cleavage activity using an F-Q reporter. NTC: no-template control. **(D)** *M. pneumoniae* fluorescence intensity compared with NTC within 10 min of reaction.

Enzymatic recombination amplification (ERA) is an isothermal amplification technology developed by GenDx Biotech Co., Ltd., in 2019 and is a modified version of RPA technology (Xia and Chen, 2020). The method retains the characteristics of specific amplification of target DNA fragments over a wide temperature range (25–42°C), and the amplification reaction time is only 15 min at the optimum temperature. Compared with RPA technology, ERA technology is more efficient, adaptable, and robust. Here, we combined the “collateral cleavage activity” of LbCas12a with ERA isothermal amplification and introduced single-stranded DNA (ssDNA) tagged with the reporter group to develop a novel *M. pneumoniae* DNA detection method, allowing observation of fluorescence using a portable fluorometer or visual readout with lateral flow assay (LFA), termed the ERA/CRISPR–Cas12a dual system (Figure 1A). This study is a great attempt to apply the system to the detection of *M. pneumoniae*, hoping to establish a rapid, economic, ultrasensitive, and specific detection method for *M. pneumoniae*.

## Materials and Methods

### Materials and Reagents

All oligonucleotides and recombinant plasmids were chemically synthesized by Shanghai Sangon Biotech (China). The QIAamp DNA Mini Kit (no. 51306) used for DNA extraction was purchased from QIAGEN GmbH (Germany). The Basic ERA Kit (no. KS101) was purchased from GenDx Biotech Co., Ltd. (China). The LbCas12a recombinant protein (no. E-002) was purchased from Shanghai HuicH Biotech Co., Ltd. (China). The HybriDetect Dipstick (no. MGHD 1) for dipstick system detection was purchased from Milenia Biotec GmbH (Germany). The commercial qPCR kit was purchased from Sansure Biotech Inc. (China). NEBuffer 2.1 was formulated according to its reagent composition (New England BioLabs, no. B7202).

### Bacterial Strains

The strains of *M. pneumoniae* (*M129*, ATCC 29342) and *Chlamydia psittaci* (*6BC*) were retained in our laboratory. The strains of *Mycoplasma pirum* (*Mpi*, ATCC 25960), *Mycoplasma hominis* (*MH*, ATCC 23114), and *Mycoplasma penetrans* (*MPe HF-2*) were donated by Hengyang Medical School, University of South China. The strains of *Staphylococcus aureus* (*SAU*, ATCC 25923), *Pseudomonas aeruginosa* (*PAE*, ATCC 27853), *Acinetobacter baumannii* (*AB K09-14*), *Klebsiella pneumoniae* (*KPN*, ATCC 700603), and *Haemophilus influenzae* (*HIB 65290_NP_Hi3*) were obtained through purchase. These strains were inoculated into the liquid medium, frozen at −80°C, which were activated by passaging, and stored at −20°C before use. All samples were stored at 4°C for 72 h before DNA extraction.

### DNA Extraction

Genomic DNA from *M. pneumoniae*, other pathogens, and clinical samples was extracted using the QIAamp DNA Mini Kit following the manufacturer’s instructions. The extracted DNA was stored at -80°C until use.

### Design of Enzymatic Recombination Amplification Primers

When making a differential diagnosis, a unique conservative sequence of *M. pneumoniae* must be selected to distinguish it from other pathogens. The most conserved sequences of *M. pneumoniae* were *ATPase*, adhesion *P1*, and the conserved region of *16SrRNA*. The sequence alignment of the adhesion *P1* gene (>NC_000912.1:180858-182404) using the online software BLAST (National Center for Biotechnology Information, NCBI) confirmed the sequence to be a conserved sequence with high homology. Primers were designed using the online software Primer-BLAST (NCBI), following the ERA primer design principles. Analytical specificity was determined using BLAST, ensuring that there were no matches with other pathogens. Details are presented in Figure 1B and Table 1.

**TABLE 1:** Primers and probes used in the ERA/CRISPR–Cas12 dual system.

Name	Sequence (5′-3′)	Length
Forward Primer 1 (F1)	AAAGAAATCGGACTCGGAGGACAATGGTCAG	31
Forward Primer 2 (F2)*	AAGAAATCGGACTCGGAGGACAATGGTCAG	30
Reverse Primer 1 (R1)	CATAAGGCGCATCGTACAGAATCAGGATCGAG	32
Reverse Primer 2 (R2)*	GTACAGAATCAGGATCGAGGCGGATCATTTGG	32
Forward Primer (F)	GACTCACCGTAGTGGGACACTTCACAAGTACCA	33
Reverse Primer (R)	GTTCGGGTGGGATCATACGTGGTTTGTTGACTG	33
F-Q	/56-FAM/TTATTATT/3BHQ_1/	8
F-B	/56-FAM/TTATTATT/3Biotin/	8

The oligonucleotide sequences of ERA primers and non-specific single-stranded DNA for the trans-cleavage assay used by the ERA/CRISPR–Cas12a dual system for the detection of M. pneumoniae. F and R were the forward and reverse primers designed for gRNA2. The remainder was primers designed for gRNA1. The final sequences are marked with an asterisk (*). F-Q was used in the fluorescence system and F-B in the dipstick system for trans-cleavage detection.

### Screening of gRNA and Reporter Probes

Generally, each mature Cas12–gRNA begins with 19 nt (nucleotides) of the direct repeat, followed by 23–25 nt of the spacer sequence. For the LbCas12a recombinant protein, the repeat sequence was 5′-UAAUUUCUACUAAGUGUAGAU-3′, which was paired with a 20-nt gRNA of the target gene as the spacer that must be located on one side of the TTTN PAM label. To obtain a more optimal guide RNA (gRNA) for *M. pneumoniae*, gRNAs targeting the *P1* gene were designed and scored using CRISPR-DT online software for Cpf1 (Cas12a). The optimal candidate scheme was obtained by considering the prediction target efficiency and miss effect, as listed in Table 2. The candidate gRNAs used in this study were synthesized by Sangon Biotech (Shanghai) Co., Ltd.

**TABLE 2:** The Sequences and the corresponding targeting efficiency scores of the gRNAs used in this study.

Name	Sequence (5′-3′)	Target efficiency Score
gRNA1*	UAAUUUCUACUAAGUGUAGAUGUACUGCCCUUUCCAGUUCG	0.9112
gRNA2	UAAUUUCUACUAAGUGUAGAUGCUACACCCGCCCUGACGAG	0.9112

The final sequences are marked with an asterisk (*).

Because the “collateral cleavage activity” of Cas12a could recognize ssDNA, the sequence 5′-FAM-TTATTATT-BHQ1-3′ was used as a fluorescence probe (F-Q) for non-specific cleavage to observe the fluorescence results. For the reporter probe of lateral flow, the F-B probe with the sequence 5′-FAM-TTATTATT-Biotin-3′ was selected.

### Establishment of ERA/CRISPR–Cas12a Fluorescence System

The rapid detection of *M. pneumoniae* using the ERA/CRISPR–Cas12a fluorescence system was completed in two steps. The first was the isothermal amplification of *M. pneumoniae* DNA using the Basic ERA Kit. The following were used for the analysis: 20 μL of dissolving agent, 2.5 μL of each of forward and reverse primers, 21 μL of ddH_2_O, 2 μL of activator, and 2 μL of template which were added to the reaction tube containing freeze-dried powder to prepare 50 μL of ERA mixtures. The centrifuged reaction tubes were placed in a constant temperature incubator at 35°C for 15 min. The LbCas12a–gRNA complex was prepared for construction. A total of 150 nM LbCas12a was preincubated with 60 nM gRNA in 5 × NEBuffer 2.1 for 10 min at 37°C, and then, the F–Q probe was added to the tube at a final concentration of 300 nM. Finally, 2 μL of the amplicons was mixed with 18 μL of LbCas12a–gRNA complex and 80 μL of 1 × NEBuffer 2.1 ready to run on the machine. Fluorescence was acquired using a fluorescence plate reader, and data were collected at 37°C for 40 cycles at 1-min intervals.

To optimize the experimental state, the whole system was explored multifaceted, including ERA reaction time and temperature, selection of gRNAs and ERA primers, and optimization of gRNA, LbCas12a, and F-Q concentrations. During system construction and optimization, 2 μL of *M. pneumoniae* standard strain with a concentration of 10^7^ copies/μL was used as the target template to determine the optimal conditions. When various reaction conditions yielded indistinguishable results, the template was diluted and reoptimized until a clear difference was obtained.

### Development of ERA/CRISPR–Cas12a Dipstick System

When the dipstick system was performed, F-Q was replaced with F-B at a final concentration of 200 nM. The 100-μL LbCas12a trans-cleavage assay was allowed proceeding for 15 min at 37°C. For each sample, 100 μL of HybriDetect Assay Buffer was pipetted into a new reaction tube, 5–10 μL of the ERA/CRISPR–Cas12a detection product was added to the solution, and the HybriDetect Dipstick was placed into the solution. Generally, 2 min was sufficient to develop a positive band at the test line.

The ERA/CRISPR–Cas12a dipstick system is developed on the basis of the fluorescence system, only the reporter probe and the incubation time of the CRISPR system are different from that of fluorescence system. Therefore, only the concentration of the F-B probe (1 nM–10 μM) and incubation time of the CRISPR–Cas12a system (5 min–1 h) were explored in the dipstick system.

### Sensitivity and Specificity of the ERA/CRISPR–Cas12a Dual System

To test the sensitivity of the ERA/CRISPR–Cas12a dual system targeting the *P1* gene of *M. pneumoniae*, the longest ERA primer pair sequence of 269 bp was intercepted and cloned into a pUC57 vector to construct a standard plasmid. The copy number of DNA molecules was calculated according to the following formula: Amount (copies/μL) = [DNA concentration (g/μL)/(plasmid length in base pairs × 660)] × 6.02 × 10^23^. These DNA samples were suspended in 1 × TE buffer, and the final concentrations were adjusted from 10^6^ to 10^0^ copies/μL as *P1* DNA standard solutions and stored at -20°C until use.

*SAU*, *PAE*, *KPN*, *HIB*, *AB*, and *Cps* were consistent with the clinical symptoms of *M. pneumoniae*, causing respiratory infections; *Mpi*, *Mpe*, and *MH* belonged to the *Mycoplasma* genus, with a similar shape and gene structure to *M. pneumoniae*. Therefore, these nine strains were used for specificity validation. Genomic DNA was extracted using the QIAamp DNA Mini Kit and stored at −20°C. Analytical specificity testing was performed using the ERA/CRISPR–Cas12a dual system for the detection of *M. pneumoniae* and other samples.

### Validation With Clinical Samples

To evaluate the ERA/CRISPR–Cas12a dual system, 92 throat swabs from children aged 3–10 years verified by qPCR were used in this study, including 56 positive samples and 36 negative samples. The swabs were obtained by trained personnel and renumbered to protect patient privacy. Total nucleic acids were extracted using QIAamp DNA Mini Kit. All assays were performed in triplicate. The positive or negative predictive agreement (PPA/NPA) of the ERA/CRISPR–Cas12a dual system for clinical samples was estimated using qPCR results as the standard.

The extracted DNA was amplified by a real-time PCR using a commercial qPCR kit, and the detection results were monitored using an ABI 7500 system. According to the instructions, 38 μL of the reaction solution, 2 μL of the enzyme mix, and 0.4 μL of the internal standard were mixed in proportion to each reaction. Ten microliters of the sample to be tested was added to each tube and allowed standing for 10 min. Take 40 μL of the mixture into each tube, centrifuge and test on the machine. The thermal cycle was performed under the following conditions: 50°C for 2 min, 94°C for 5 min, then 45 cycles at 94°C for 15 s, 57°C for 31 s, and finally 25°C for 10 s.

## Results

### Feasibility Analysis of the ERA/CRISPR–Cas12a Fluorescence System

We assessed the feasibility of the ERA/CRISPR–Cas12a fluorescence system for *P1* gene detection. Figure 1C shows that at the initial stage of the reaction, the fluorescence intensity of *M. pneumoniae* was greatly increased and tended to plateau at approximately 20 min, with a value of more than 2 × 10^6^, and it was 22 times that of the negative control, which was extremely different from that of the negative control. Subsequently, the fluorescence intensity of *M. pneumoniae* did not increase anymore and maintained a straight line, revealing that the reaction reached equilibrium. These results demonstrated that the reaction speed of the ERA/CRISPR–Cas12a fluorescence system was extremely rapid and reached a saturation state in approximately 20 min. It was deduced that this system could accurately cut and detect the *P1* gene of *M. pneumoniae*, and the LbCas12a–gRNA complex could generate “collateral cleavage” activity and shear the introduced reporter probe after it was activated. In addition, the results for *M. pneumoniae* were also significantly different from the negative sample within 10 min of reaction (Figure 1D), allowing approximate results to be observed in 10 min or even 5 min.

**FIGURE 2:**
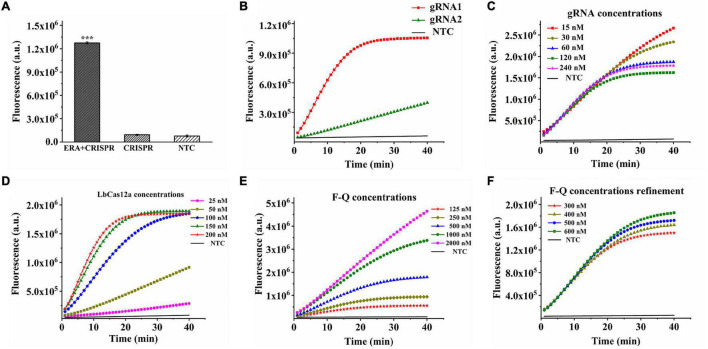
Optimization of CRISPR–Cas12a system in ERA/CRISPR–Cas12a fluorescence system. **(A)** The importance of ERA amplification for CRISPR system detection. ERA + CRISPR: detection results of the whole reaction system; CRISPR: sample without ERA amplification and directly tested with the CRISPR system. Error bars in panels represent the mean ± SD, where *n* = 3 replicates. The optimized fluorescence intensity of the system on reaction screening of gRNAs **(B)**, gRNA concentration **(C)**, LbCas12a concentration **(D)**, and F-Q concentration **(E,F)**, respectively.

Additionally, when the samples had no ERA amplification and only participated in CRISPR–Cas12a detection, there was no change in fluorescence intensity and no obvious difference compared with the negative control (*p* > 0.05) (Figure 2A), indicating the importance of combining the CRISPR–Cas12a system with ERA technology to improve the sensitivity of this system.

### Optimization of the ERA/CRISPR–Cas12a Fluorescence System

Adhesion protein *P1* is highly conserved for *M. pneumoniae*, here, two gRNAs were designed for this gene, and the results showed that gRNA1 reached the plateau within 20 min, while gRNA2 reacted incompletely after 40 min and continued to cleave. The fluorescence intensity was much lower than that of gRNA1 (Figure 2B), indicating better cleavage efficiency of gRNA1. Therefore, the gRNA1 complementary sequence was regarded as the optimal target sequence. After gRNA selection, we continued to explore the concentrations of gRNA, LbCas12a, and F-Q for the CRISPR–Cas12a system. By observing the fluorescence curves generated by the concentrations of different gRNAs and LbCas12a proteins, it was found that the CRISPR reaction was completed in approximately 20 min when the gRNA concentration reached 60 nM. The reaction reached a plateau phase, and the concentration of LbCas12a protein combined with gRNA to form a complex was 150 nM (Figures 2C,D). In addition, various concentrations of the fluorescence probe were introduced into the reaction. When the concentration of the F-Q probe was 500 nM, the reaction reached equilibrium in a shorter time, and the fluorescence intensity was significantly higher than that of the low-concentration probes (Figure 2E). Figure 2F shows that 300–600-nM F-Q probes produced consistent results, therefore, 300 nM was chosen as the optimal F-Q probe concentration.

**FIGURE 3:**
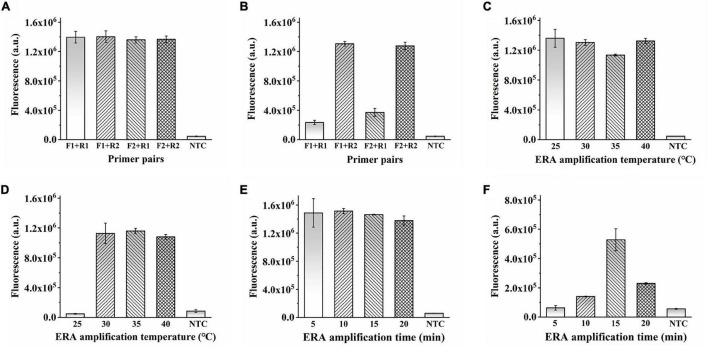
Optimization of ERA in ERA/CRISPR–Cas12a fluorescence system. **(A,B)** Optimization of ERA primer. Template concentration: 10^7^ copies/μL **(A)**, 10^5^ copies/μL **(B)**. **(C,D)** Optimization of ERA temperature. Template concentration: 10^7^ copies/μL **(C)**, 10^5^ copies/μL **(D)**. **(E,F)** Optimization of ERA time. Template concentration: 10^5^ copies/μL **(E)**, 10^2^ copies/μL **(F)**. Error bars in panels represent the mean ± SD, where *n* = 3 replicates.

In addition to optimizing the CRISPR–Cas12a system, the ERA amplification reaction must be explored. Four primer pairs were used to determine the optimal ERA reaction primers. As detected by the ERA/CRISPR–Cas12a fluorescence system, no obvious differences were observed when the sample concentration was sufficient (Figure 3A). When the template concentration was reduced to 10^5^ copies/μL, the four primer pairs produced different amplifications (Figure 3B). Therefore, the forward primer 2 combined with the reverse primer 2 (F2 + R2), with the highest fluorescence intensity, was selected as the best primer pair. Similarly, when the template concentration was sufficient, it had the same amplification ability at 25–40°C. When the template concentration reduced to 10^5^ copies/μL, the fluorescence intensity generated at the ERA reaction temperature of 25°C was consistent with the results of the negative samples, whereas the other three temperatures still had higher fluorescence; here, 35°C was chosen as the optimal reaction condition (Figures 3C,D).

It is worth noting that the amplification appeared to be already successful at 5 min for the ERA reaction time as shown in Figure 3E. That is because the ERA/CRISPR–Cas12a system constructed in this study is a secondary signal amplification system. When the amount of template was high enough, the whole reaction system was subjected to 5 min of ERA amplification, which had generated a certain amount of product that could be detected by the CRISPR system recognition, and the corresponding fluorescence was generated (Figure 3E); when the template concentration was reduced to 10^2^ copies/μL, the product produced by ERA amplification for 5 min cannot meet the detection limit of the CRISPR system, less fluorescence or even no fluorescence was generated, whereas the fluorescence intensity at 15 min was much higher than that under other conditions (Figure 3F). The fluorescence intensity decreased after 15 min because the CRISPR–Cas12a system detection was based on ERA amplification, so the results generated were affected by ERA technology. With a large amount of template, the dNTPs were continuously consumed and reacted with magnesium ions in the ERA system to form a large amount of insoluble magnesium pyrophosphate solution, and the solution became turbid. The part of the amplicon was subsequently transferred to the CRISPR–Cas12a system, which was also correspondingly turbid, resulting in a decrease in fluorescence intensity. Therefore, 15 min was considered the optimal reaction time for ERA. In this study, the ERA technique could exhibit the best amplification for 15-min reaction at 35°C for rapid detection by the CRISPR–Cas12a system.

### Visualization of the ERA/CRISPR–Cas12a Dipstick System

**FIGURE 4:**
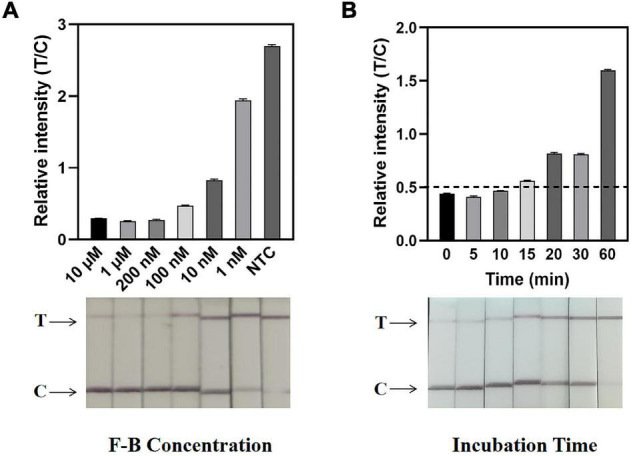
Determination of F-B concentration and reaction time by the ERA/CRISPR–Cas12a dipstick system. **(A)** Concentration optimization of the dipstick probe (F-B). The relative intensities of the band are shown at the top, and the dipstick results for different F-B concentrations are shown below. **(B)** An optimal incubation time of the CRISPR–Cas12a system. The black horizontal dashed line in the gray scan indicates the cutoff value set based on T/C values of the negative control. Error bars in panels represent the mean ± SD, where *n* = 3 replicates.

**FIGURE 5:**
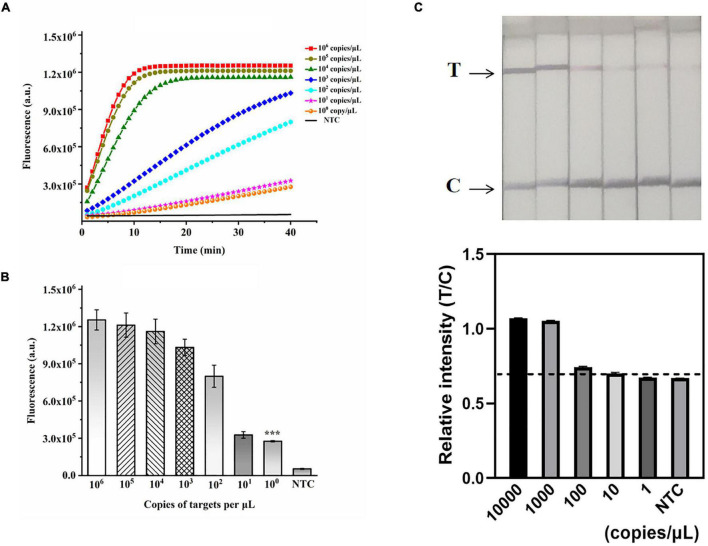
Validation of the sensitivity of the ERA/CRISPR–Cas12a dual system. **(A)** Fluorescence curves generated by the ERA/CRISPR–Cas12a fluorescence system at each dilution. **(B)** Comparison of fluorescence values generated after 40 min of ERA/CRISPR–Cas12a fluorescence system reaction at each dilution. ***p<0.001. **(C)** Analytical sensitivity of the ERA/CRISPR–Cas12a dipstick system at each dilution. The black horizontal dashed line in the gray scan indicates the cutoff value set based on T/C values of the negative control. Error bars in panels represent the mean ± SD, where *n* = 3 replicates.

**FIGURE 6:**
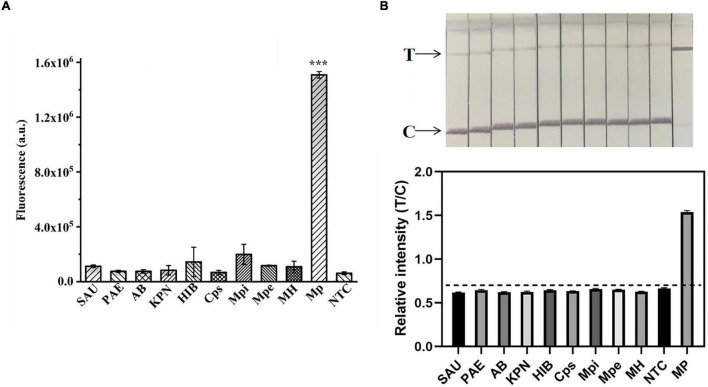
Validation of the specificity of the ERA/CRISPR–Cas12a dual system. Specificity test of the ERA/CRISPR–Cas12a fluorescence system **(A)** and dipstick system **(B)** for targeting *M. pneumoniae*. *SAU*: *Staphylococcus aureus*; *PAE*: *Pseudomonas aeruginosa*; *AB*: *Acinetobacter baumannii*; *KPN*: *Klebsiella pneumoniae*; *HIB*: *Haemophilus influenzae*; *Cps*: *Chlamydia psittaci*; *Mpi*: *Mycoplasma pirum*; *MPe*: *Mycoplasma penetrans*; *MH*: *Mycoplasma hominis*. The black horizontal dashed line in the gray scan indicates the cutoff value set based on T/C values of the negative control. Error bars in panels represent the mean ± SD, where *n* = 3 replicates. ***P < 0.001.

**FIGURE 7:**
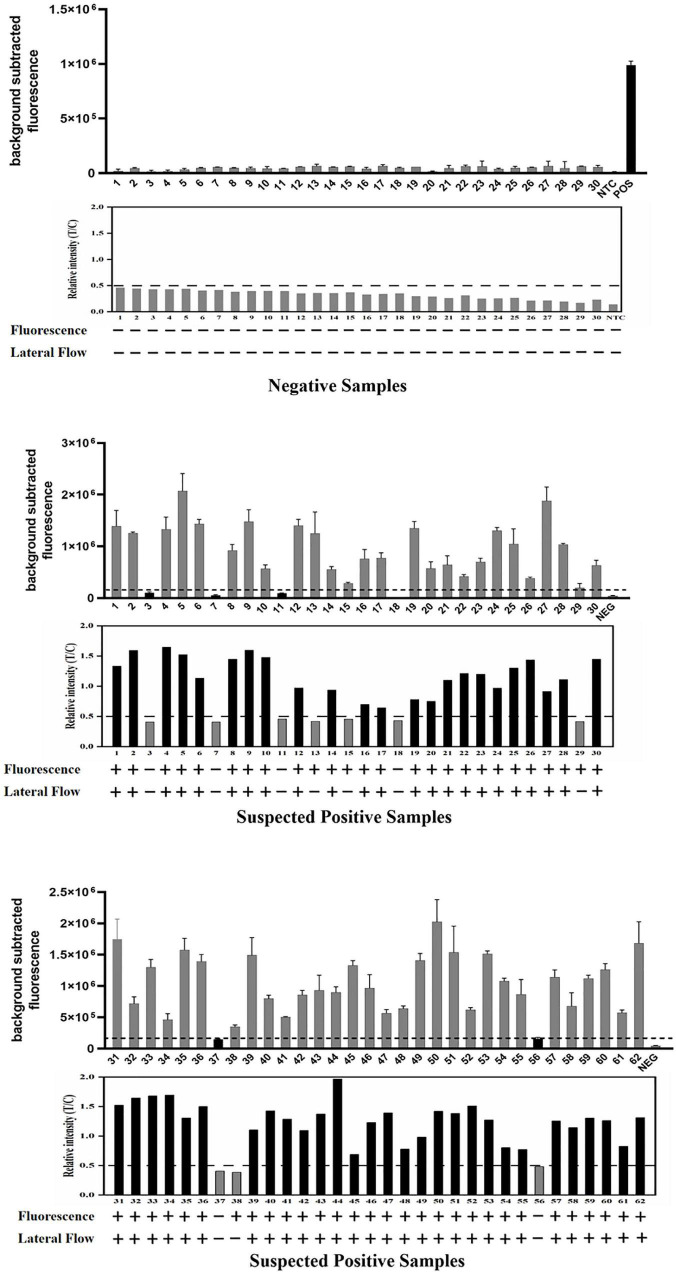
Detection of 92 clinical samples by the ERA/CRISPR–Cas12a dual system for *M. pneumoniae*. For each group of results, the detection results of the ERA/CRISPR–Cas12a fluorescence system are displayed at the top, the gray scan results of the strips are displayed in the middle, and the bottom is the interpretation results of the dual system. Background subtracted fluorescence was the fluorescence intensity of the experimental group against the blank control. Black horizontal dashed line indicates the threshold for a positive result. + : indicates a positive sample, −: indicates a negative sample. Error bars in panels represent the mean ± SD, where *n* = 3 replicates.

The nearly complete extinction of the T-line is the basis for an easy and intuitive interpretation of test strip results. The interpretation of the HybriDetect Dipstick was summarized in the supplementary material of Broughton et al. (2020), which stated that T-line results alone could also be interpreted as positive results, and slight T-lines did not affect the negative result interpretation. Additionally, to avoid errors caused by subjective factors, the band gray levels on the dipsticks were scanned and the relative intensity (T/C) ratio was calculated in this study. The T/C ratio of the negative controls plus 2SD was set as the threshold for the LFA results, which are represented by the black dotted line in the Figures 4–7.

Defining a specific reporter concentration is important for easily interpretable readout and sensitive LFA performance. To eliminate the T-line intensity as much as possible, it was necessary to determine the required amount of the F-B probe in the ERA/CRISPR–Cas12a dipstick system. Dipsticks were placed directly into F-B dilutions containing different concentrations, with the lowest probe concentration, at which the T-line disappeared as the optimal concentration. From the dipstick visual results, the intensity of the T-line produced by the F-B concentration of 200 nM was significantly lower than that of the C-line. From the gray-scale scanning results, the T/C ratio of the F-B probe at a concentration of 200 nM was relatively low, which was basically the same as that at 10 μM. Thus, 200 nM was determined to be the optimal probe concentration for the dipstick system (Figure 4A).

The incubation time of the CRISPR–Cas12a system in the dipstick system was also explored and optimized. The reaction tubes were incubated at 37°C for 5 min, 10 min, 15 min, 20 min, 30 min, and 1 h, respectively (Figure 4B). The results showed that the T/C ratio of the dipstick incubated for 15 min was greater than the threshold, and there was an obvious difference between the positive and negative strips. Therefore, the optimal incubation time of the dipstick system was 15 min.

### Analytical Sensitivity and Specificity of the ERA/CRISPR–Cas12a Dual System

A recombinant plasmid of the *M. pneumoniae P1* gene was constructed to verify the sensitivity of the ERA-CRISPR/Cas12a dual system. The plasmid was serially diluted to final concentrations of 10^6^, 10^5^, 10^4^, 10^3^, 10^2^, 10^1^, and 10^0^ copies/μL and used as templates for dual-system detection. *M. pneumoniae* DNA was detected by the ERA/CRISPR–Cas12a fluorescence and dipstick systems at concentrations down to 1 copy/μL (Figures 5A,B) and 100 copies/μL (Figure 5C), respectively. In particular, the sensitivity of the fluorescence system was comparable to that of the commercial qPCR kit, demonstrating an ultrahigh sensitivity; the dipstick system was slightly lower, but it did not need to rely on fluorescence instruments and could be used in areas lacking health resources.

To explore the ERA/CRISPR–Cas12a dual-system specificity, we used 10 strains for validation. The fluorescence results showed that only *M. pneumoniae* was statistically significant compared with the negative control (*p* < 0.001) (Figure 6A), and the dipstick results also showed that only *M. pneumoniae* had an obvious T-line, and the gray scanning ratio was higher than the threshold (Figure 6B). Our results showed that the specificity of the dual system was 100% and that there was no cross-reactivity with other pathogens. In short, the ERA/CRISPR–Cas12a dual system is ultrasensitive and specific for potential use in clinical samples.

### Validation of the ERA/CRISPR–Cas12a Dual System With Clinical Samples

We evaluated the performance of the ERA/CRISPR–Cas12a dual system on 92 specimens. To define the positive samples for fluorescence readout, we set the signal-to-noise ratio parameter (the ratio of the fluorescence value of the sample to the negative control, S/N) to S/N > 3 after 40 min of the fluorescence system reaction, which was considered a positive sample (Ding et al., 2021). The detection results of the fluorescence system are displayed at the top, the gray scanning results of the dipstick system are shown below and correspond one by one, and the dual-system detection results of each sample are listed at the bottom. The PPA and NPA of the ERA/CRISPR–Cas12a fluorescence system relative to the commercial qPCR kit were 100%, and those of the ERA/CRISPR–Cas12a dipstick system were 92.86% and 100%, respectively (Figure 7 and Table 3). The ERA/CRISPR–Cas12a dual-system clinical sample validation results were no worse than those of the commercial qPCR kit and showed excellent clinical applicability.

**TABLE 3:** Comparison between the performance of the ERA/CRISPR–Cas12a dual system and commercial qPCR kit.

	ERA/CRISPR–Cas12 Fluorescence System		ERA/CRISPR–Cas12 Dipstick System		Total (*n* = 92)
	Positive	Negative	Positive	Negative	
Positive	56	0	52	4	56
Negative	0	36	0	36	36
PPA/NPA	PPA: 100%	NPA: 100%	PPA: 92.86%	NPA: 100%	

*Data were presented as the number of specimens.*

## Discussion

The detection of *M. pneumoniae* is essential for the control of primary atypical pneumonia. In recent years, the rapid detection methods based on the CRISPR–Cas system have been developed and applied to various pathogens, such as *SARS-CoV-2, African swine fever virus, MERS-CoV*, *SARS-CoV*, *Yersinia pestis, Mycobacterium tuberculosis, Salmonella*, and *Pseudomonas aeruginosa* (Bai et al., 2019; Ali et al., 2020; Mukama et al., 2020; Talwar et al., 2021; Wang et al., 2021; Xia et al., 2021; You et al., 2021; Zhang et al., 2021). However, the application of the CRISPR–Cas12a system to detect *M. pneumoniae* has not yet been established. Therefore, in this study, we developed an ERA/CRISPR–Cas12a dual system that can diagnose the *M. pneumoniae P1* gene with ultrahigh sensitivity and specificity and does not cross-react with other pathogens. The entire assay can be performed within 30 min with a low reagent cost and simple instrument use, making this system an attractive option to develop point-of-care diagnostic tests for disease control.

The ERA real-time fluorescence method was previously used by our group to detect *M. pneumoniae* with a sensitivity of 100 copies/μL. In this study, we combined ERA basic amplification technology with the CRISPR–Cas12a system, explored the reaction conditions, comprehensively analyzed the whole experimental results from multiple angles, and finally determined the best reaction system with single-copy sensitivity (1 copy/μL), which was two orders of magnitude higher than that of the ERA real-time fluorescence method. Compared with other methods for the rapid detection of *M. pneumoniae*, such as RAA and LAMP (Arfaatabar et al., 2019; Xue et al., 2020), the ERA/CRISPR–Cas12a fluorescence system exhibited higher sensitivity. In addition, the sensitivity was equivalent to a concentration of 1.67 aM, which was comparable to the sensitivity of DETECTR (Chen et al., 2018). And this study also showed higher sensitivity compared with other methods constructed based on the CRISPR–Cas12a system, such as iSCAN-OP with femtomolar sensitivity (Aman et al., 2020) and LAMP-coupled CRISPR–Cas12a system with a concentration of 100 aM (Mahas et al., 2021). Therefore, the ultrahigh sensitivity of our ERA/CRISPR–Cas12a system shows significant advantages for clinical detection.

In addition, the ERA/CRISPR–Cas12a dual system constructed in this study can detect *M. pneumoniae* DNA within 30 min. The ERA amplification only took 15 min, the incubation time of the dipstick detection system was 15 min, and the fluorescence system reached a plateau in approximately 20 min; therefore, the ERA/CRISPR–Cas12a dual system only took 30 min to detect *M. pneumoniae*. Furthermore, *M. pneumoniae* DNA extraction could be completed within 25 min, and the time from samples to results of *M. pneumoniae* was approximately 1 h, which was generally consistent with the reaction time of the CRISPR–HBV system (Chen et al., 2021) and Cas12a–DETECTR assay (Ding et al., 2021), and was much lower than the 3-h detection time of Cas12a/gRNA trans-cleavage fluorescence assay for MTB detection (Xu et al., 2020). In general, the ERA/CRISPR–Cas12a dual system fulfills the need for rapid nucleic acid detection.

The results of this study were very consistent with those of qPCR in the analysis of clinical outcomes. The results of using commercial qPCR and the ERA/CRISPR–Cas12 fluorescence system to detect *M. pneumoniae* in 92 clinical samples were in complete agreement. The PPA and NPA of the dipstick system and qPCR were 92.86% and 100%, respectively, which were comparable to the clinical applicability of *SARS-CoV-2* DETECTR and ITP-enhanced CRISPR assays (Broughton et al., 2020; Curti et al., 2021). In addition, the system was affordable, with a dipstick system of US$ 8/reaction and a fluorescence system of only US$ 4/reaction, which was lower than that of the Lyo-CRISPR SARS-CoV-2 kit (US$ 12/reaction) (Curti et al., 2021). Finally, experimental equipment of the ERA/CRISPR–Cas12a dual system did not require complicated instrumentation, and the ERA amplification temperature had a wide applicability range (25–40°C) and could be incubated in a small thermostat or water bath pot. If it was in hot weather or lacked resources, it was directly placed on the table for 15 min. The dipstick system permitted direct visualization of the results; the detection of the fluorescence system utilized a portable fluorometer, which was suitable for base site detection.

Although the ERA/CRISPR–Cas12a dual system has the advantages of ultrahigh sensitivity and specificity, short time, and economic convenience, the following aspects should be further refined in future research. The clinical sample size involved in this study was small, which should be increased for further validation. The sensitivity of the LFA system should be improved, and the background interference on dipsticks should be decreased. The presented system currently requires two steps and can be evolved into a single-tube reaction. Besides, this system can be developed into a nucleic acid extraction-free system to further combine with artificial intelligence to reduce human resources. In conclusion, we have developed an ERA/CRISPR–Cas12a dual system with ultrasensitivity, good specificity, speed, short time, and low cost for *M. pneumoniae* detection. This system not only meets the need for on-site rapid detection in resource-limited areas, but is also more effective in avoiding the delayed therapy of *M. pneumoniae* infection due to delayed etiologic diagnosis, in terms of both respiratory and extra-respiratory complications.

## Data Availability Statement

The datasets presented in this study can be found in online repositories. The names of the repository/repositories and accession number(s) can be found below: https://www.ncbi.nlm.nih.gov/, ATCC29342.

## Ethics Statement

This study was approved by the Human Ethics Committee of Affiliated Nanhua Hospital, University of South China (approval date: 8 Jan 2021; approval number: 2021-ky-181), and complied with the Declaration of Helsinki.

## Author Contributions

ZD, HH, and JH built the framework and wrote the manuscript. HH, XS, DT, and JL collected and sorted the materials. TJ, XH, WY, DZ, and XX contributed with literature support. All authors contributed to the revision and accepted version of the manuscript.

## Conflict of Interest

The authors declare that the research was conducted in the absence of any commercial or financial relationships that could be construed as a potential conflict of interest.

## Publisher’s Note

All claims expressed in this article are solely those of the authors and do not necessarily represent those of their affiliated organizations, or those of the publisher, the editors and the reviewers. Any product that may be evaluated in this article, or claim that may be made by its manufacturer, is not guaranteed or endorsed by the publisher.
